# Sexual Health of Patients in Treatment for Lung Cancer: An Undercover Concern for Patients and Oncologists

**DOI:** 10.3390/curroncol32020107

**Published:** 2025-02-13

**Authors:** Mafalda Costa, Catarina Lopes Fernandes, Joana Leite, Marta Vilaça, Fernanda Estevinho, Helena Magalhães

**Affiliations:** Medical Oncology Department, Pedro Hispano Hospital, 4464-513 Matosinhos, Portugal; catarina.lopesfernandes@ulsm.min-saude.pt (C.L.F.); joana.basilioleite@ulsm.min-saude.pt (J.L.); marta.vilaca@ulsm.min-saude.pt (M.V.); fernanda.estevinho@ulsm.min-saude.pt (F.E.)

**Keywords:** lung cancer, sexual health, survey, patient-reported outcome, quality of life

## Abstract

Sexual dysfunction (SD) prevalence in lung cancer (LC) patients is largely unknown. This study aims to assess the prevalence of SD among LC patients at our center. We conducted a cross-sectional survey of 69 patients using a questionnaire on sexual activity and satisfaction. Participants were recruited from 1 July 2023 to 30 September 2024 and had to be diagnosed with LC, age > 18 years, able to read, and with at least 1 month of treatment. A total of 61 patients completed the survey, predominantly male (67.2%) with stage IV LC (68.9%). Less than half (45.9%) reported recent sexual activity, while many expressed little to no interest (55.7%) and minimal satisfaction with their sex life (42.8%). The factors affecting sexual satisfaction included fatigue (37.7%) and feelings of anxiety/stress (24.6%). The reasons for decreased sexual activity included a lack of interest (36.4%), difficulties with erection (24.2%), and issues with partners (24.3%). A significant association was found between SD and the perceived impact of LC on sexual life, with higher dysfunction scores linked to more negative reports. SD is common among LC patients. Implementing assessment strategies and interventions may improve the sexual life of these patients.

## 1. Introduction

Lung cancer is the most common cancer for men and the second most common for women. With 2.4 million new cases worldwide in 2022, lung cancer has the highest incidence of all cancer diagnoses [1]. Most patients are diagnosed in advanced stages, with 60 to 70% having stage IV disease at diagnosis [2]. The recent introduction of targeted therapies and immunotherapy, particularly checkpoint inhibitors, has significantly improved patient prognosis, resulting in a 5-year survival rate ranging from 15% to 50% depending on the biomarker [3,4,5]. Furthermore, the introduction of additional treatment strategies, such as consolidation and maintenance treatments, has increased the length of time patients undergo treatment. As more patients are living longer with lung cancer, interest in their quality of life during long-term systemic treatment is becoming more prominent. Additionally, the impact of the disease and treatment on the social, psychological, and physiological aspects of patients’ lives has grown into a considerable concern in recent years for physicians caring for these patients [6,7]. Despite the recent advances in lung cancer treatment and longer survival rates, patients with this condition continue to experience significant symptoms compared to other cancer patients. Throughout their cancer journey, these individuals face numerous challenges, not only related to the disease itself but also due to the various treatment modalities used, from the initial diagnosis to the later lines of treatment. Both the disease and its treatment burden can have a considerable impact on the sexual health of these patients. The prevalence of sexual dysfunction varies depending on the type of cancer, and the specific treatment modalities employed, including surgery, systemic therapy, radiotherapy, stereotactic radiosurgery, and stereotactic body radiation therapy [8]. Many questions about sexual health in cancer patients are often overlooked in clinical practice. Most studies on sexuality in this population primarily focus on breast, gynecological, or prostate cancers, often misapplying conclusions to other cancers [9]. This narrow focus can lead to incorrect assumptions about patients’ needs to be carried out from patients with different cancers, who experience very distinct treatments and surgeries. A study evaluating the effects of lung cancer treatment on sexual life found that 51.7% of the patients had concerns related to their sexual life [10]. Various factors, both directly and indirectly linked to the oncologic diagnosis, can affect the quality of sexual life. Symptoms of lung cancer, such as pain, fatigue, and dyspnea, can contribute to sexual dysfunction [10]. Additionally, the diagnosis and treatment can cause psychological stress for both the patient and his partner, which may further compromise the couple’s sexual quality of life. The high prevalence of depression and anxiety among these patients, combined with the impact of surgery and chemotherapy, contributes to fatigue and other symptoms that intensify psychological stress—ultimately significantly affecting sexual function [11,12]. During the cancer journey, sexual dysfunction is often not discussed between patients and physicians, creating a barrier to the proper recognition and management of this issue [13]. Research on sexual health, specifically with lung cancer patients, is scarce. Most studies were conducted before the advent of immunotherapy and targeted therapies, leaving the actual impact of these treatments on patients’ sexual lives and the prevalence of sexual dysfunction largely unknown. With a growing number of long-term survivors, there is an unmet need across the sexual domain and its impact on patients with lung cancer. The SHAWL (Sexual Health Assessment in Women with Lung Cancer) was the first study aiming to understand the real impact of diagnosis and treatment on the sexual health of women with lung cancer. Approximately 77% of the patients who responded to the survey reported a total disinterest in any sexual activity [14]. The primary reasons cited for this disinterest included fatigue, sadness, issues with their partner, and dyspnea [14]. By gaining a deeper understanding of the real prevalence of sexual dysfunction in patients with lung cancer, new attitudes and interventions may emerge in clinical practice, significantly improving both the patients’ sexual health and quality of life. In this study, we aim to evaluate the prevalence of sexual dysfunction among lung cancer patients undergoing systemic treatment at our center and assess its impact on their quality of life. Additionally, we seek to identify the potential factors related to the disease and its treatments.

## 2. Materials and Methods

This was an observational cross-sectional study on patients with a lung cancer diagnosis at any stage performed at the Medical Oncology department of our center, Hospital Pedro Hispano. Participant recruitment started on 1 July 2023 and finished on 30 September 2024. Eligibility criteria included lung cancer diagnosis, age > 18 years old, ability to read, and patients had to have completed at least 1 month of systemic treatment and suspended it in less than 3 months. Patients with disabling psychiatric conditions, who were unable to give consent and to read or write were not considered for the survey. An opportunistic sampling was performed by the investigators of this study, and the informed consent and survey forms were given to potential candidates on paper at their Medical Oncology appointment if they accepted to participate. A coding system was used to identify the questionnaires and maintain confidentiality, and the participants returned the form to their physician when completed. Clinical information regarding patient diagnosis and treatments, comorbidities, habits, and other demographic characteristics were obtained from clinical reports. The final authority on content was the principal investigator. The survey’s questionnaire was specifically designed for lung cancer patients by the study team and revised by all the investigatory team members. It consisted of a male and female version of a multiple-choice questionnaire about sexual activity and satisfaction. The survey asked about sexual activity pre-lung cancer diagnosis and over the “past 30 days” before the inclusion of the patient in the study. The survey was written in the patient’s native language, Portuguese. It included 15 multiple-choice questions and 3 yes/no questions with a total of 18 questions in its female version and 15 multiple-choice and 4 yes/no questions with 19 questions in its male version, respectively (Appendix A.1 and Appendix A.2—Portuguese version, and Appendix B.1 and Appendix B.2—English translation). The authors decided to adopt a total of 10 questions from the two validated questionnaires the PROMIS Sexual Function and Satisfaction (PROMIS SexFS brief profile) not translated to Portuguese and The EORTC Sexual Health Questionnaire in Portuguese (Portugal) adapting it to the special difficulties of lung cancer patients according to their experience, and using terms and language more understandable by the population treated at our center. A total of 7 questions created by the team members were added to the male and the female versions not for measuring purposes of sexual dysfunction, but to evaluate the patient’s perception of lung cancer diagnosis impact on sex life, evaluate problems with sex life before diagnosis, and acknowledge the communication gap in our population around these topics. As these questions are for demographic characterization, we did not perform a new validation of the questionnaire.

The statistical analysis was performed using SPSS version 29.0. Descriptive analysis was performed using absolute and relative frequencies for categorical variables and means and standard deviation for continuous variables when presented with a normal distribution, or median and quartiles instead when the data did not have a normal distribution. Skew and Kurtosis coefficients were considered as the Shapiro–Wilk test and quartile diagram observation to evaluate the normal distribution. We created two scores to assess different aspects of sexual health. The first score measures sexual dysfunction and is based on the sum of points from questions 5 to 10 adapted from the validated questionnaires mentioned above. The second score evaluates the patient’s perception of the impact of a lung cancer diagnosis on their sex life and satisfaction, derived from the sum of points from questions 12 to 14. For the female version of the questionnaire, the scoring for questions 7 to 10 was reversed before conducting the statistical analysis. Similarly, in the male version, the scoring for questions 5 to 10 was also reversed. This adjustment ensures that higher scores indicate a greater level of symptoms or problems with their sex life. There was no need to reverse the scoring for the second evaluated score. Since the distribution of these scores was not normal, we used non-parametric tests in conjunction with other variables, in association with Spearman coefficients, Mann–Whitney tests, and Kruskal–Wallis tests. For categorical variables, the Pearson Chi-Square test was performed. The significance level set was 5%. Regarding the rest of the questionnaire, questions 1 and 2 were scored from 1 to 5 to assess interest and sexual desire, with higher scores indicating greater interest and desire. Question 3 addressed sexual activity in the past 30 days and was conditional. The patients who reported no sexual activity proceeded to question 4, while those who reported sexual activity skipped directly to question 5. Question 15 was also conditional, requiring the patients who did not discuss any sexual health concerns with their physician to answer a related follow-up question about possible reasons for it. Only two multiple-choice questions allowed the participants to select more than one option: question 4, which addressed reasons for not having sexual activity, and question 11, which explored the factors affecting participant satisfaction with their sex life. To account for sexual health problems in the past, the participants were asked about medication used to enhance sexual performance and history of sexual dysfunction before their lung cancer diagnosis.

## 3. Results

A total of 80 patients were screened between 1 July 2023 to 30 September 2024 and 69 patients agreed to participate in the study. Only 61 participants answered a minimum of ⅔ of the questions included in the final report and eight questionnaires were excluded from the analysis. The majority of the participants were men (67.2%) with an ECOG performance status of 1 (60.7%). Most patients had stage IV lung cancer (LC) (68.9%) and 19.7% were on treatment in the context of a relapse. The patients presented a median time on the current treatment of 5.3 months and were mainly on treatment with immunotherapy (24.6%) followed by tyrosine kinase inhibitors (TKIs) (21.3%) and chemoimmunotherapy (19.7%). Table 1 describes patients, Table 2 lung cancer characteristics, and Table 3 treatment characteristics.

### 3.1. Sexual Activity

Within the prior 30 days, less than half the patients (n = 28, 45.9% with a mean age of 61.7) had reported sexual activity. Regarding interest in sexual activity, the majority of the patients (n = 34, 55.7%) reported little to no interest, and 44.3% of the patients (n = 27) reported never or rarely wanting to have sexual activity. When we analyzed the results by sex, we found that 51.2% of the males and 35% of the females reported having engaged in sexual activity. Additionally, 53.7% of the males and 70% of the females indicated that they had little to no interest in sexual activity. Furthermore, 31.4% of the males and 60% of the females stated that they rarely or never want to engage in sexual activity.

**Figure 1:**
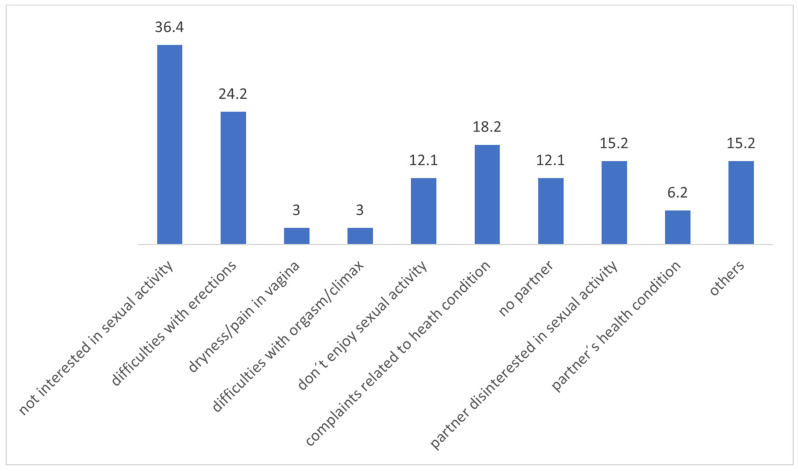
Graph with the patient-reported reasons for not having sexual activity in the last 30 days; the participants could choose more than one option as this was a multiple-choice question. Presented in the graph are relative frequencies.

In question number 4, which asked the participants about the reasons for not engaging in sexual activity over the past month, the most commonly selected options were as follows: 36.4% reported a lack of interest in sexual activity, 24.2% mentioned difficulties with erection, 18.2% cited complaints related to health condition, and 12.1% stated that they did not enjoy sexual activity. These data are illustrated in Figure 1.

Other reasons stated by the participants that were not among the available options included fatigue (n = 3), dyspnea (n = 3), sadness (n = 3), lack of motivation (n = 1), and the impact of the disease (n = 1).

### 3.2. Sexual Satisfaction

Out of the 28 patients who reported engaging in sexual activity in the past 30 days, 42.8% expressed that they were either not at all satisfied or only slightly satisfied with their sex life. Among these, 47.6% of the men and 28.6% of the women reported this level of dissatisfaction. Additionally, 23.8% of the men experienced little to no pleasure from their sex life, compared to only 14.3% of the women. Regarding the ability to achieve orgasm or climax, approximately 47.6% of the men and 57.1% of the women reported that they could do so rarely or sometimes. When they were able to reach orgasm, about 28.6% of the men and 42.9% of the women reported feeling some level of satisfaction from the experience.

Only 14.3% of the men were able to maintain an erection much more than half the time during sexual intercourse. Among women with sexual activity, 17.8% experienced some level of pain or discomfort inside their vagina and 14.3% felt some level of discomfort in their genitals during sexual intercourse.

### 3.3. Sexual Activity Before Cancer Diagnosis

Concerning the impact of LC diagnosis on sexual activity, 28 patients (45.9%) reported that their sex life changed somewhat to very significantly after the diagnosis. Among the women, 35% noted that their sexual experiences were somewhat to very different from before the diagnosis, while 34.1% of the men reported the same change. A total of 30 patients (49.2%) revealed that their diagnosis had some to a lot of impact on their interest in sexual activity, and also 35 participants (57.4%) considered that their cancer diagnosis had some to a lot of impact on the quality of their sex life. When analyzed by sex, 51.2% of the men and 45% of the women reported experiencing some to a lot of impact from their diagnosis on their interest in sexual activity. Additionally, 65.9% of the men and 55% of the women reported a similar level of impact on the quality of their sex lives. An association between sexual dysfunction and the patient-reported impact of their diagnosis on sexual life and satisfaction was found suggesting that the patients with higher sexual dysfunction scores (score sum of questions nº 5–10) were also the ones considering that LC had an impact on the subject (score sum of questions nº 12–14) (rs = 0.54, *p* = 0.006).

When asked about some of the possible factors that may have influenced satisfaction with their sex life in question number 11, some patients selected mostly emotional factors related to their oncological diagnosis such as feeling anxious, stressed, or angry (24.6%) and also feeling depressed, sad, or unhappy (16.4%). Other participants chose factors related to lung cancer symptoms such as fatigue (37.7%) and dyspnea or cough (16.4%), among others seen in Table 4. Additionally, some participants stated factors related to treatment secondary effects such as alterations in sensibility (14.8%), and gastrointestinal changes (6.6%).

In question number 16, 23% of the patients (n = 14) stated already having problems in their sex life before LC diagnosis, and among them 42.9% were women. Also, 8.2% of the men used medication in the past to improve their sexual performance. Only 11.5% of the patients talked to their physician about changes in sexual activity and satisfaction with their sex life, mostly because they thought it was not important to talk about it (29.5%) or did not feel comfortable talking about it (11.5%).

### 3.4. Subgroup Analysis and Risk Factors for Sexual Dysfunction

Two scores were performed, one for sexual dysfunction (score sum of questions nº 5–10) and the other for the patient’s perception of the impact of lung cancer diagnosis on their sex life and satisfaction (score sum of questions nº 12–14). These scores were used for subgroup analysis and to study the possible associations between treatment and disease characteristics with sexual health.

Regarding sexual activity within the prior 30 days, 65% of the women (n = 13) versus 48.8% of the men (n = 20) did not have any sexual activity (*p* = 0.233). Difficulties with erection were reported by 40% of the men who indicated not having engaged in sexual activity in the past 30 days (n = 20) and only 1 woman reported dryness or pain in or around the vagina as the reason for not having any sexual activity in the last 30 days. Women reported less interest and desire to have sexual activity with lower median scores when questioned about sexual interest (median score 2 vs. 3, on a scale of 1–5; *p* = 0.029) and sexual desire (median score 2 vs. 3, on a scale of 1–5; *p* = 0.011). The men and women had no differences regarding sexual dysfunction score (median score 20 vs. 16; on a scale of 4–30; *p* = 0.671) and also in the reported impact of LC in their sex life (median score 9 vs. 7.5, on a scale of 3–15; *p* = 0.227), although the men tendentially presented higher scores.

**Figure 2:**
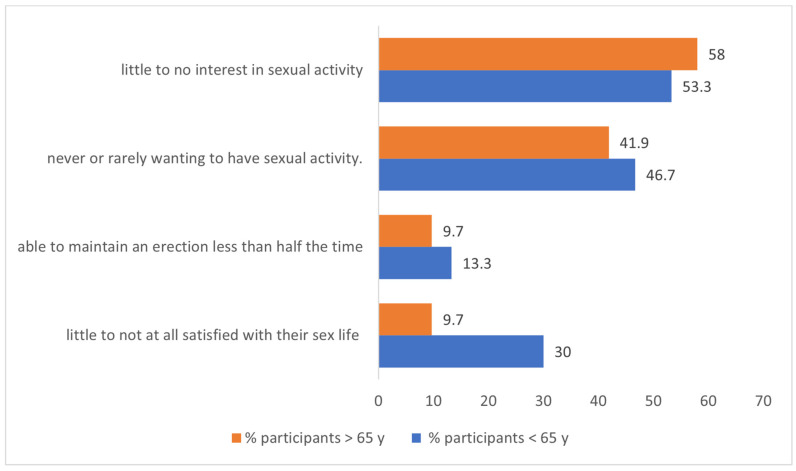
Graph describing little to no interest in recent sexual activity, never or rarely wanting to have sexual activity, able to maintain erection less than half the time, and little to not at all satisfied with their sex life, stratified by age groups (>65 y, n = 31; <65 y, n = 30).

No associations were found between higher scores for sexual dysfunction (rs = 0.095, *p* = 0.652) and higher scores for the impact of LC diagnosis in sex life with the participants’ age (−0.106, *p* = 0.442). Around 32.3% of the participants 65 years old or older stated having sexual activity in the last 30 days compared to 60% of their counterparts (*p* = 0.030). No differences were found regarding interest and desire to have sexual activity between the age groups (<65 years and ≥65 years) by median score. Figure 2 shows the differences in sexual activity, sexual interest and desire, and sexual satisfaction between the age groups. Also, no differences regarding sexual dysfunction (median score 18.5 vs. 12.5, on a scale of 4–30; *p* = 0.315) and also in the reported impact of LC in their sex life (median score 8.5 vs. 7, on a scale of 3–15; *p* = 0.378) were found between the age groups.

**Figure 3:**
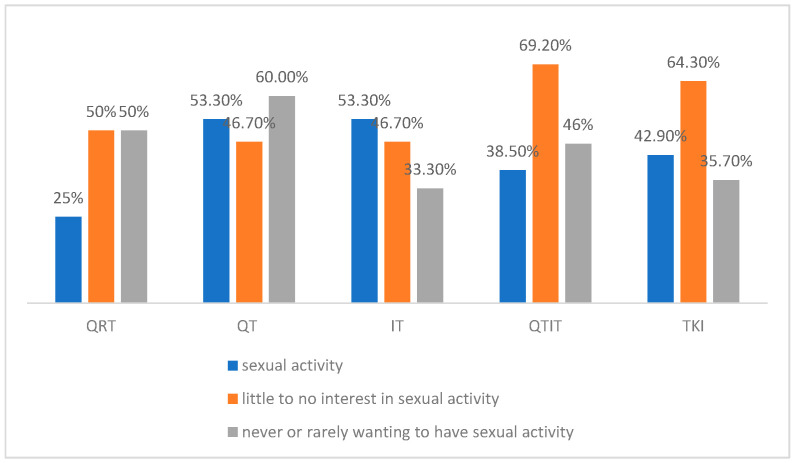
The graph represents sexual activity rate, little to no sexual interest, and never or rarely wanting to have sexual activity rate by ongoing systemic treatment. QRT: chemoradiotherapy, QT: chemotherapy, IT: immunotherapy, QTIT: chemoimmunotherapy, TKIs: tyrosine kinase inhibitors.

Some treatment-related factors that may also contribute to sexual dysfunction were explored. No difference was found between sexual activity and the type of systemic treatment ongoing (*p* = 0.765). The same happened for sexual interest (*p* = 0.486) and sexual desire (*p* = 0.480). Regarding the sexual dysfunction score and the reported impact of LC on sex life score, no association was found between the type of ongoing systemic treatment and score results (*p* = 0.246 and *p* = 0.577). Figure 3 represents sexual activity rate, little to no sexual interest, and never or rarely wanting to have sexual activity rate by ongoing systemic treatment. Also, no association was found between the line of treatment, the intention of treatment, and the number of previous treatments with sexual activity, sexual interest, sexual desire, and the two scores.

**Figure 4:**
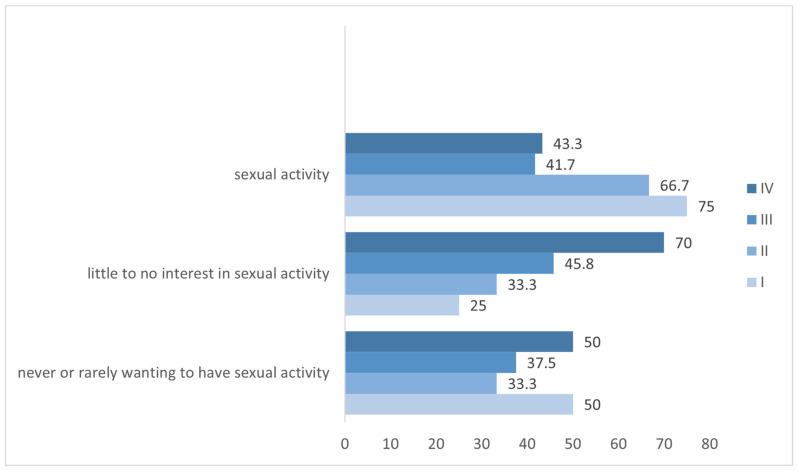
Graph showing little to no interest in recent sexual activity, never or rarely wanting to have sexual activity, and sexual activity by stage group (I, II, III, and IV).

Regarding other treatment modalities, brain radiosurgery (SRS) did not interfere with sexual activity and the two scores (*p* = 0.581 and *p* = 0.387). The participants who performed holocranial RT showed a tendency for higher scores on sexual dysfunction (median score 24 vs. 15, on a scale of 4–30; *p* = 0.071), but no influence in sexual activity, sexual interest, and sexual desire. The participants submitted to surgery presented higher sexual dysfunction scores (median score 20.5 vs. 14, on a scale of 4–30; *p* = 0.307) and higher impact of LC diagnosis on sex life score (median score 9 vs. 7, on a scale of 3–15; *p* = 0.150), but no differences were found in sexual activity (*p* = 0.164), sexual interest (*p* = 0.324), and sexual desire (*p* = 0.647). The subgroup with brain metastasis showed a tendency for higher sexual dysfunction scores (median score 20 vs. 15, on a scale of 4–30; *p* = 0.663), but no differences in sexual activity, sexual interest, and sexual desire. No association was found between the disease stage and sexual activity and satisfaction, and the two scores, as seen in Figure 4.

## 4. Discussion

This study examined 61 patients with lung cancer (LC) at various stages of the disease, all undergoing systemic treatment. Our findings revealed that 45.9% (n = 28) of the participants engaged in sexual activity in the month before the survey, with a notable gender difference: 35% (n = 7) of the women reported sexual activity compared to 51.2% (n = 21) of the men. The reasons for the lack of sexual activity included disinterest (36.4%), issues with partners (21.2%), difficulties with erections (24.2%), and complaints related to health condition (18.2%). Among those who were sexually active, 42.8% (n = 12) expressed being little to not at all satisfied with their sex life. The participants identified several factors influencing their sexual quality of life such as fatigue (37.7%), feelings of sadness or unhappiness (24.6%), dyspnea (16.4%), and changes in sensibility (14.8%). Although limited studies have been published on this topic, recent research suggests that approximately 45% of LC patients are sexually active [15]. A Spanish study, LUDICAS, found comparable rates of sexual activity among men (45%) and women (36.2%), aligning with our report of 51.2% for the men and 35% for the women [15]. The SHAWL study focused solely on women with lung cancer, and reported even higher rates of sexual activity at 54%, although it noted similar disinterest levels, with 77% of the participants expressing little to no interest in sexual activity compared to 70% in our study [14]. These studies included patients from different countries, each influenced by distinct demographics and external factors that might affect results. In our population, the factors impacting sexual health were consistent with those found in the SHAWL study, highlighting fatigue, anxiety, sadness, and dyspnea as considerable influences on sexual life quality. Notably, our female participants reported fewer issues with pain or discomfort in the vagina during sexual activity, at 17.8%, compared to 56% in the SHAWL study [15]. There are also differences in systemic treatment, with fewer female patients on targeted therapy (TKI)—40% in our study versus 47% in the SHAWL study [15]. There were disparities in sample sizes, with only 20 female patients in our study, making direct comparisons inappropriate [15]. Our findings demonstrate that emotional distress—such as anxiety or depression—substantially impacts sexual health among LC patients. We advocate for systematic assessments to identify the psychological and pharmacological interventions needed during LC treatment. Many of the factors identified can be addressed in routine clinical practice. For example, partner-related problems can often be managed through couples therapy and couple-based interventions. Additionally, strategies to alleviate treatment-related side effects, such as employing neuroleptics to manage sensibility changes, can be implemented. Addressing erectile dysfunction and vaginal dryness may also benefit from a multidisciplinary approach involving urologists, sexologists, and gynecologists to enhance sexual satisfaction. While some influencing factors, like treatment-related fatigue or self-image issues, are beyond our control, psychological support remains essential, especially in the palliative care setting where these effects may endure. Moreover, communication gaps between patients and healthcare providers hinder the effective management of sexual health concerns. Our survey revealed that only 34.4% of the participants had not discussed sexual health with their physician due to a lack of complaints. Others stated they did not consider it important (29.5%) or felt uncomfortable discussing it (11.5%), with only 11.5% actively talking about their sex life issues with their physician. This underscores the need to enhance patients’ understanding of the potential impacts of cancer and treatment on sexual function. A significant barrier arises from healthcare professionals feeling ill equipped to address sexual health, stemming from inadequate training and the absence of established interdisciplinary protocols [16,17]. As for patients, in our study, 13.8% refused to discuss the topic, despite being given the chance to complete an anonymous questionnaire. While most reported no difficulty in completing the survey (80.3%), we eliminated eight questionnaires due to inappropriate answers. Half of the participants acknowledged changes in their sex lives since their LC diagnosis, with over half believing their diagnosis significantly impacted their sexual quality of life. Women and men have distinct needs regarding sexual health, which are likely due to the well-documented anatomical and physiological variations influencing sexual dysfunction and pleasure in men and women [18]. Our study highlights the differing sexual health needs, suggesting that tailored interventions could improve care. Identifying specific sexual health issues will pave the way for introducing more targeted interventions in clinical practice. Further investigations, like the CLARIFY project and the SHAWL study, which focus on female patients, could provide valuable insights into the sexual health of different patient subgroups [14,19]. Additionally, understanding how prior medications, existing comorbidities, and lifestyle factors such as smoking influence sexual dysfunction is crucial [20,21]. Our findings indicated that 23% of the patients had sexual issues even before their LC diagnosis, with 8.2% of the men using medication to enhance sexual performance. Notably, while sexual interest often persists into older age—evidenced by 57% of the individuals aged 65–74 remaining sexually active [22]—only 32.3% of our lung cancer patients over 65 reported being sexually active. This study challenges the assumption that sexual activity decreases with age and highlights the need for careful evaluation of sexual health in younger patients, with around 40% of the young patients being sexually inactive. Moreover, LC survivors may face early sexual dysfunction challenges, particularly those who undergo surgery or chemotherapy [23]. Research indicates an increased risk of erectile dysfunction post-surgery, reinforcing the importance of early discussions about sexual health. Young patients undergoing chemotherapy may experience reduced libido, complicating their sexual function during treatment [24]. It is vital to assess their concerns and provide tailored support throughout this difficult period. With lung cancer incidence rising among younger, non-smoking populations [25], we must not make assumptions about sexual dysfunction based solely on age. Unfortunately, our study did not identify significant associations between ongoing systemic treatments and sexual dysfunction, potentially due to the underrepresentation of certain treatment subgroups. The same is true for disease characteristics like cancer stage and the presence of brain metastasis, limiting our ability to draw meaningful conclusions. Future research should focus on the impact of specific treatments, such as immunotherapy and TKIs, on sexual health, as limited information currently exists. Investigating specific patient groups—including those receiving treatment for brain metastases, undergoing lung surgery, or chemoradiotherapy—will be crucial for uncovering subgroup-specific sexual concerns. Clinicians must enhance their understanding of how lung cancer diagnosis and treatment affect sexual health to effectively address these patients’ needs. A multimodal approach involving various healthcare professionals—urologists, gynecologists, psychologists, physiotherapists, and psychiatrists—will be essential to provide targeted interventions. Additionally, raising awareness and providing training in communication regarding oncosexuality among healthcare providers is vital. In conclusion, utilizing questionnaires can help promote open communication between physicians and patients on this sensitive topic, ultimately improving the care of lung cancer patients and addressing their sexual health concerns effectively.

### Strengths and Limitations

This study presented some limitations to its design. Patient selection relied on the caring physician to choose the participants, which may introduce potential selection bias. Those who declined to answer the survey might have had more serious sexual health issues and opted to avoid the topic, despite the anonymity of the questionnaire. The study also faced limitations due to its cross-sectional design—it does not assess sexual health continuously throughout the cancer care continuum nor address the changes that may occur during and after treatment. The opportunistic sampling of the survey could lead to the underrepresentation of certain groups. Additionally, there were small sample sizes for certain treatment subgroups and disease characteristics, such as cancer stage and the presence of brain metastasis, limiting the ability to draw meaningful conclusions about those specific populations. Due to the underrepresentation of specific treatments like holocranial radiotherapy and stereotactic radiosurgery (SRS), the study could not conclude about their impact on sexual health. The validation of the questionnaire adapted for LC patients was not performed as it was adapted from pre-existing validated questionnaires, such as the EORTC-Sexual Health Questionnaire (EORTC SHQ-22) available in Portuguese and the PROMIS SexFS brief profile. The questionnaire was designed to specifically address issues related to LC and was tailored to our Portuguese population, taking advantage of the team’s clinical experience addressing this specific population’s concerns, which we view as a strength of the study. Some multiple-choice questions helped us identify the factors linked to sexual inactivity, influences on sexual satisfaction, and reasons for not discussing these issues with healthcare professionals. In addition, questions regarding the perceived impact of diagnosis on sexual function are very important to assess our population’s awareness of these issues. Additionally, this study included patients receiving novel therapies for lung cancer with potentially different impacts on patient’s sex life. Although the EORTC-Sexual Health Questionnaire (EORTC SHQ-22) is available in Portuguese, its original validation study did not include Portuguese patients and it was not specifically tailored for lung cancer patients, as only a small percentage (5.2%, n = 9) was included in its phase 3 design [26]. Further studies are essential in the Portuguese population to explore sexual health issues among LC patients, and a nationwide study would be of interest. This research sheds light on the sexual health concerns of LC patients and highlights significant issues.

## 5. Conclusions

Our study highlights the prevalence of sexual health problems among lung cancer patients, which are often overlooked in daily practice. There is a significant lack of communication between patients and physicians regarding this issue, preventing the identification of specific problems and the implementation of tailored supportive care measures through coordinated referral processes for a multidisciplinary approach. We believe that the training of healthcare professionals and the education of patients are key factors for improving supportive care in this area. It is essential to gain a better understanding of the specific concerns related to sexual health in lung cancer patients. This understanding will allow us to address specific factors influencing sexual health—such as disease symptoms, treatment side effects, and partner-related issues—through a multimodal approach to offer appropriate management advice for all patients.

## Figures and Tables

**Table 1:** Patient’s characteristics. ECOG PS: Eastern Cooperative Oncology Group performance status; COPD: chronic obstructive pulmonary disease.

Patient Characteristics	
age (median)	66
sex (male)	41 (67.2%)
ECOG PS (0 and 1)—n (%)	56 (91.8%)
former smokers—n (%)	24 (39.3%)
active smoking—n (%)	20 (32.8%)
hypertension—n (%)	28 (45.9%)
diabetes mellitus—n (%)	9 (14.8%)
stroke or heart attack—n (%)	3 (4.9%)
peripheral arterial occlusive disease—n (%)	4 (6.6%)
COPD or emphysema—n (%)	16 (26.3%)
anxiety or depression—n (%)	3 (4.9%)
medication associated with sexual dysfunction—n (%)	24 (39.3%)

**Table 2:** Lung cancer characteristics. NSCLC: Non-Small Cell Lung Cancer; NOS: not otherwise specified; SCLC: Small Cell Lung Cancer; nº: number.

Lung Cancer Characteristics	n (%)
NSCLC adenocarcinoma—n (%)	38 (62.3%)
NSCLC squamous cell—n (%)	12 (19.7%)
NSCLC-adenosquamous—n (%)	1 (1.6%)
NSCLC NOS—n (%)	2 (3.3%)
Stage I-II—n (%)	5 (9.4%)
Stage III—n (%)	14 (26.4%)
Stage IV—n (%)	34 (64.2%)
SCLC—n (%)	8 (13.1%)
Stage IV—n (%)	8 (100%)
Relapse—n (%)	12 (19.7%)
nº of metastatic sites (median)	2 (1–4)
Time from diagnosis (median, months)	11 (2–126)

**Table 3:** Treatment characteristics with all treatment modalities. TKIs: tyrosine kinase inhibitors; SRS: stereotactic radiosurgery; RT: radiotherapy; SBRT: stereotactic body radiation therapy.

Systemic Treatment		Radiotherapy		Surgery	
Immunotherapy—n (%)	15 (24.6%)	brain SRS—n (%)	4 (6.6%)	lobectomy—n (%)	6 (9.8%)
TKI—n (%)	13 (21.3%)	holocranial RT—n (%)	11 (18.0%)	atypical lung resection—n (%)	2 (3.3%)
Chemotherapy—n (%)	15 (24.6%)	Chemoradiotherapy—n (%)	23 (37.7%)	bilobectomy—n (%)	1 (1.6%)
Chemoimmunotherapy—n (%)	13 (21.3%)	SBRT/RT (lung and others)—n (%)	6 (9.8%)	brain surgery—n (%)	2 (3.3%)
Bispecific antibodies—n (%)	1 (1.6%)	antalgic RT—n (%)	3 (4.9%)		
Chemoradiotherapy—n (%)	4 (6.6%)				
Curative intent—n (%)	19 (31.1%)				
Line of treatment ≥ 2—n (%)	11 (18%)				
Time of ongoing treatment (median, months)	5.3				
Time since the beginning of treatment (median, months)	9.4				

**Table 4:** Patient-reported factors interfering with their sex life; the participants could choose more than one option as this was a multiple-choice question. alt: alterations; GTI: gastrointestinal.

Emotional Factors		Disease-Related Factors		Treatment-Related Factors	
anxiety, stressed, or angry	15 (24.6%)	fatigue	23 (37.7%)	alt. sensibility	9 (14.8%)
depressed, sad, or unhappy	10 (16.4%)	dyspnea or cough	10 (16.4%)	GTI changes	4 (6.6%)
		weight loss	2 (3.3%)	medical device (port or oxygen)	2 (3.3%)
		any pain	1 (1.6%)	hair loss	1 (1.6%)

**Figure A1:**
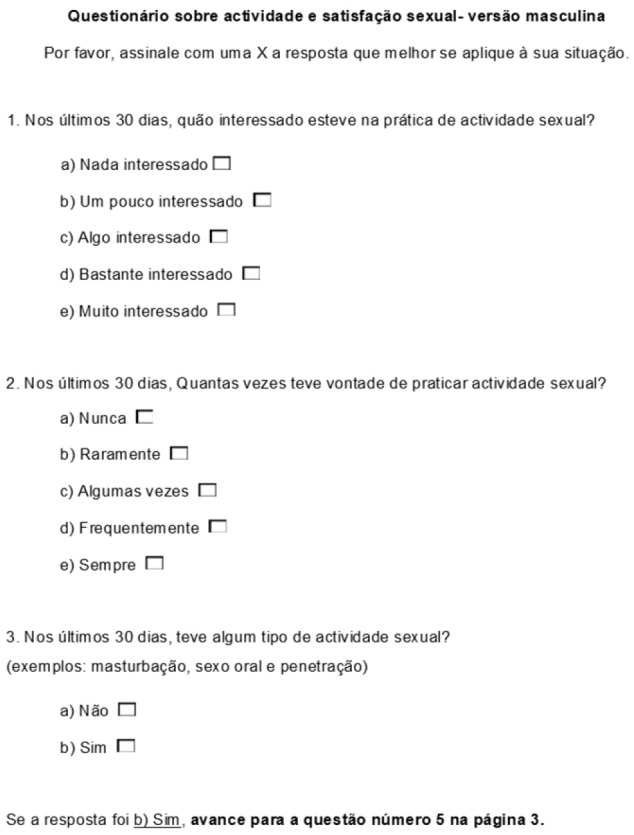
Page one.

**Figure A2:**
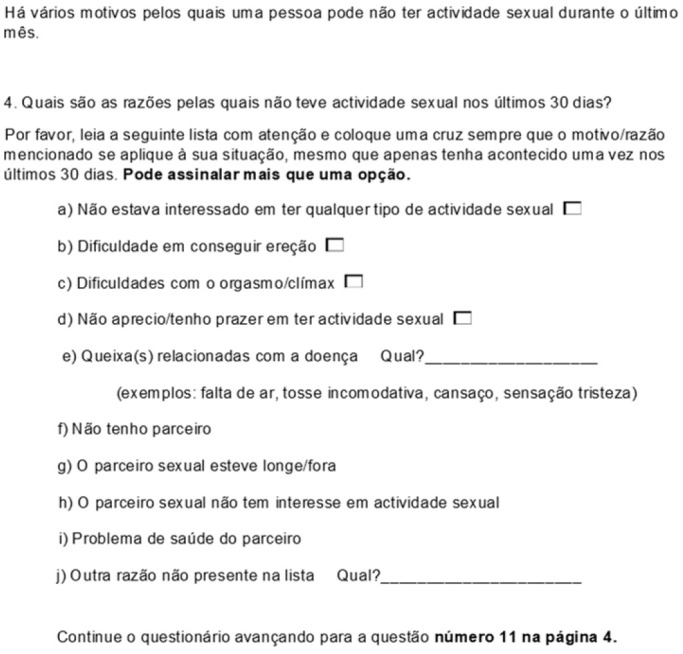
Page two.

**Figure A3:**
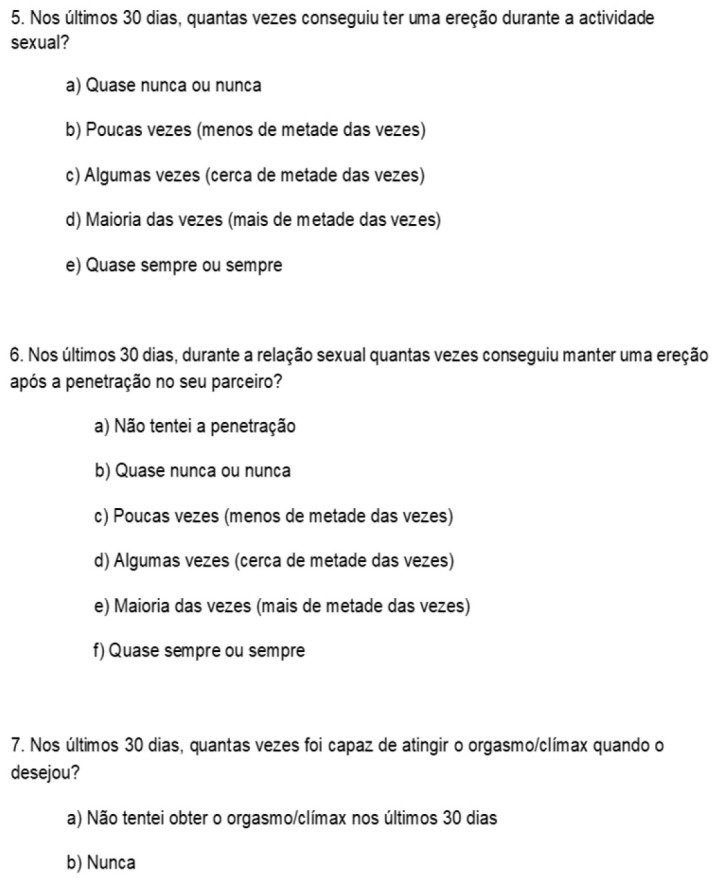
Page three.

**Figure A4:**
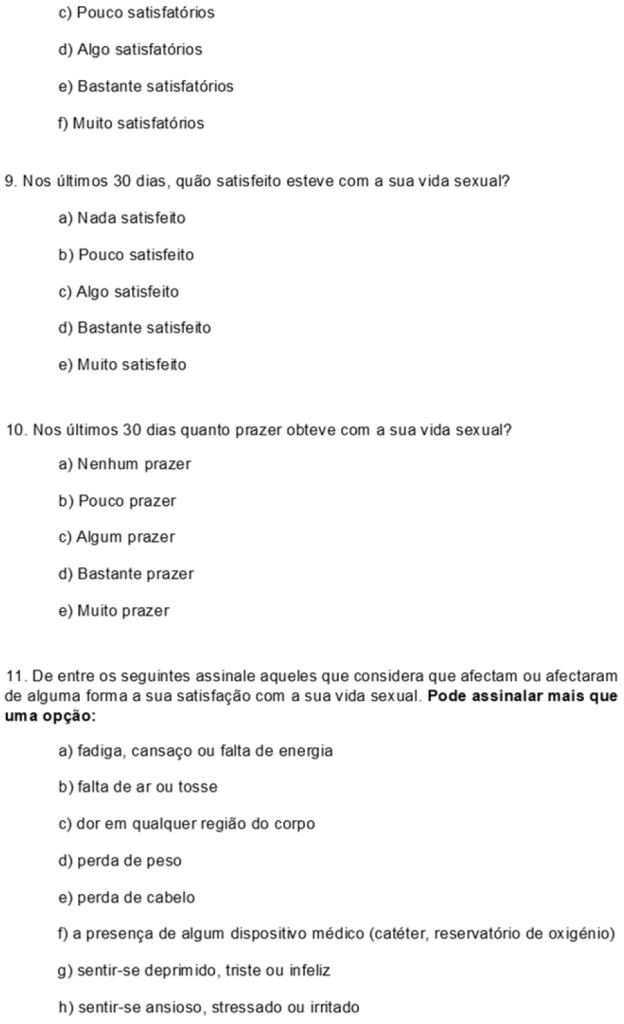
Page four.

**Figure A5:**
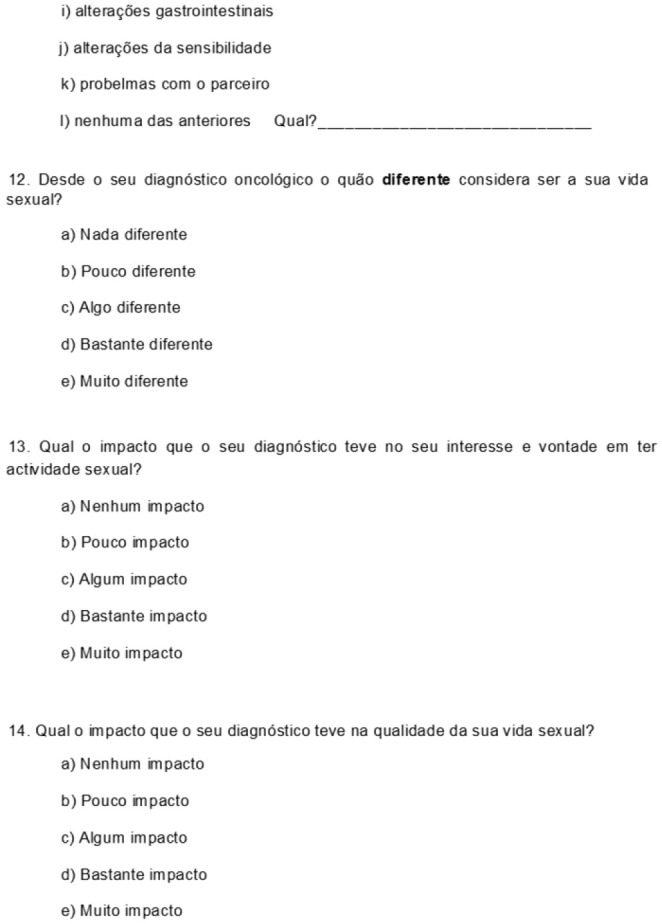
Page five.

**Figure A6:**
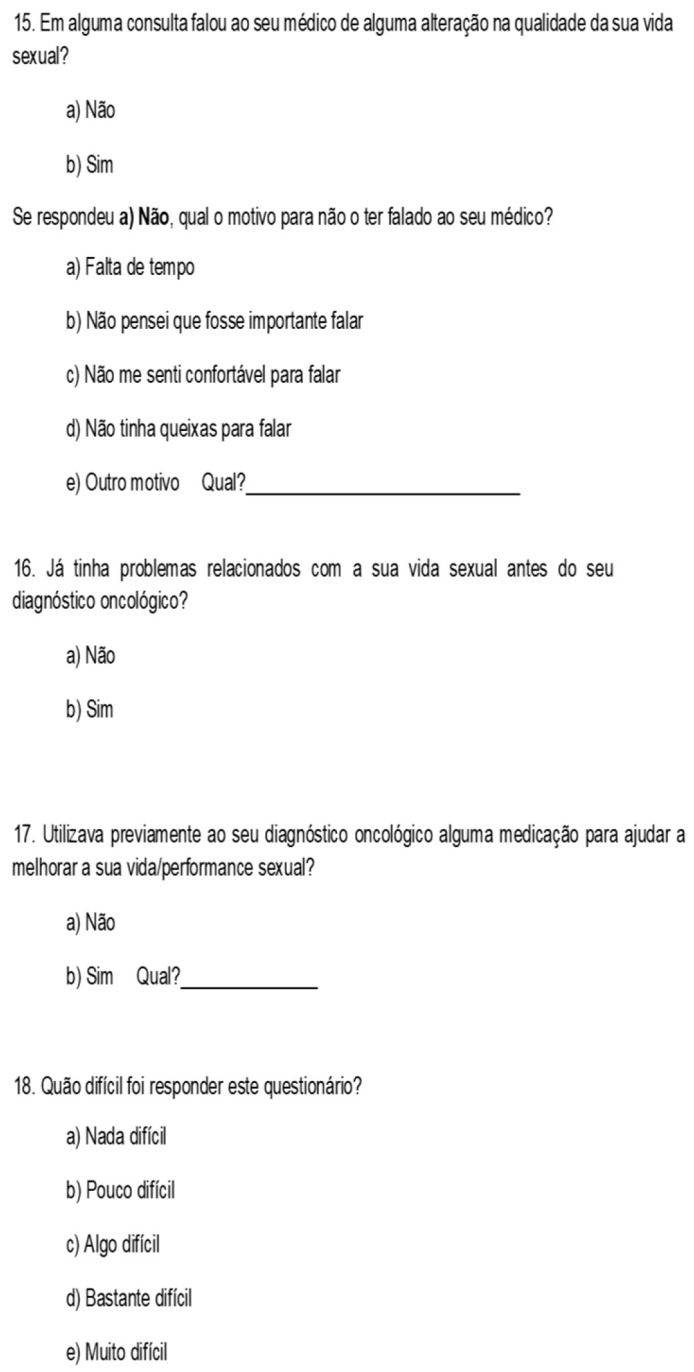
Page six.

**Figure A7:**
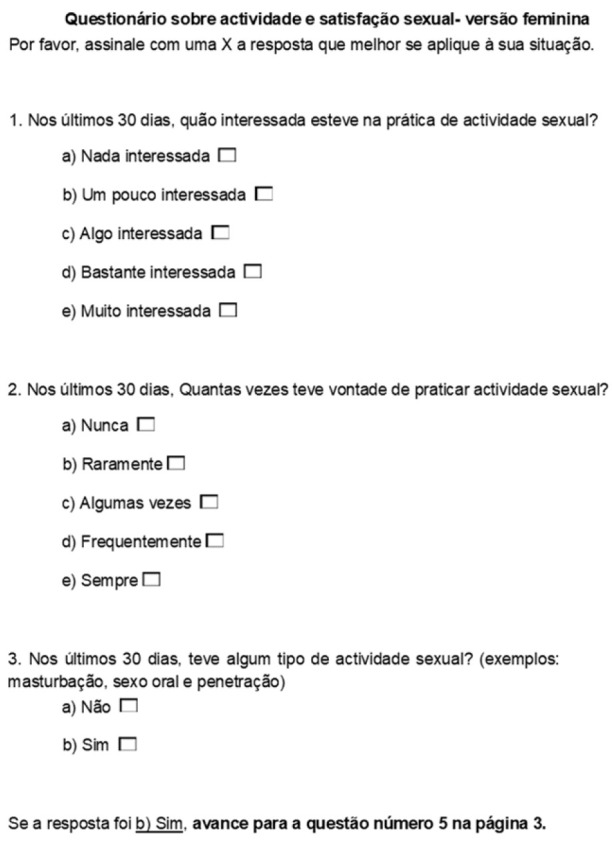
Page one.

**Figure A8:**
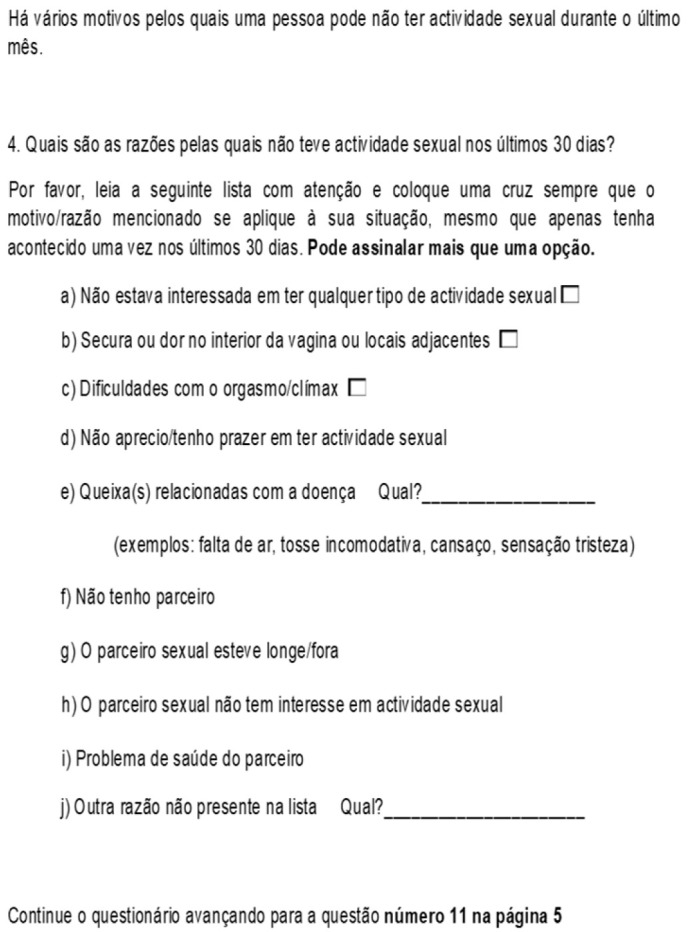
Page two.

**Figure A9:**
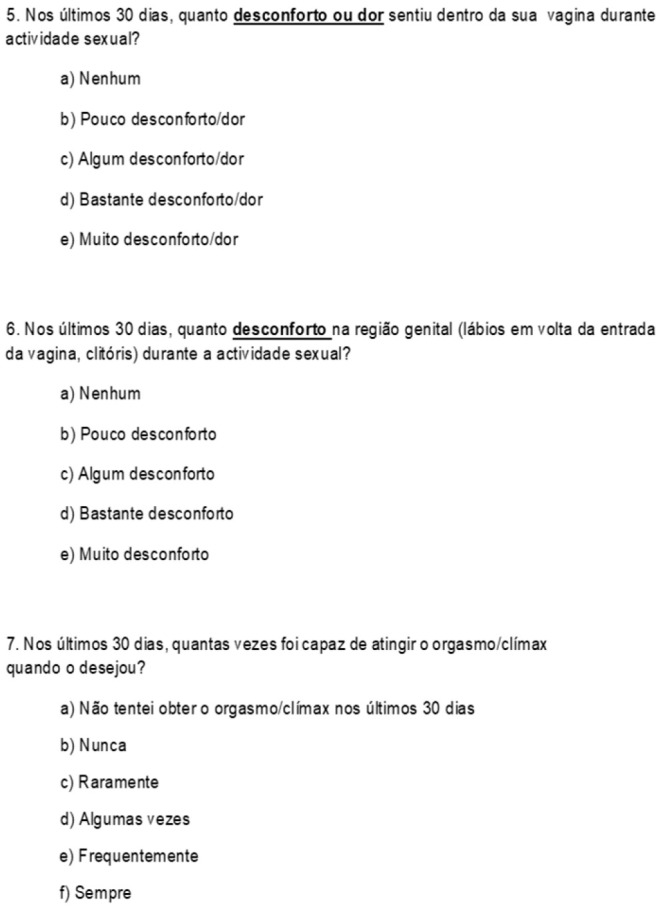
Page three.

**Figure A10:**
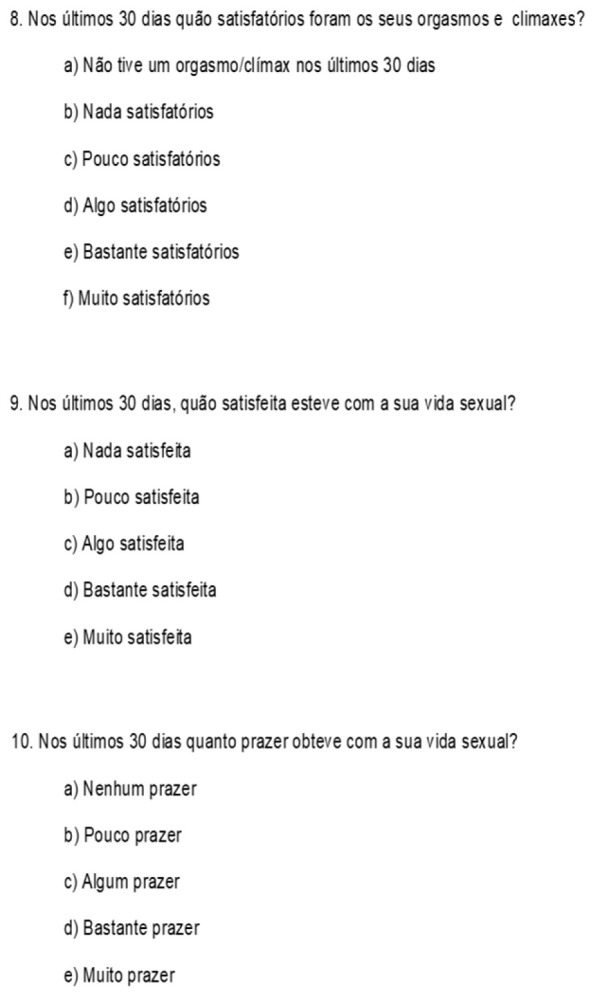
Page four.

**Figure A11:**
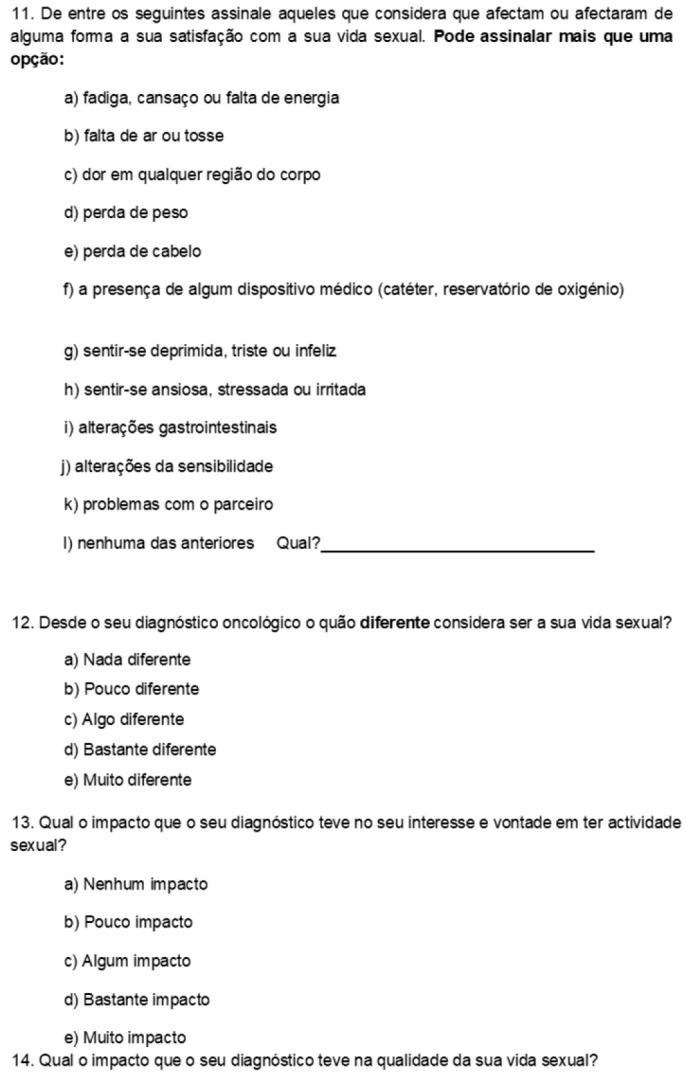
Page five.

**Figure A12:**
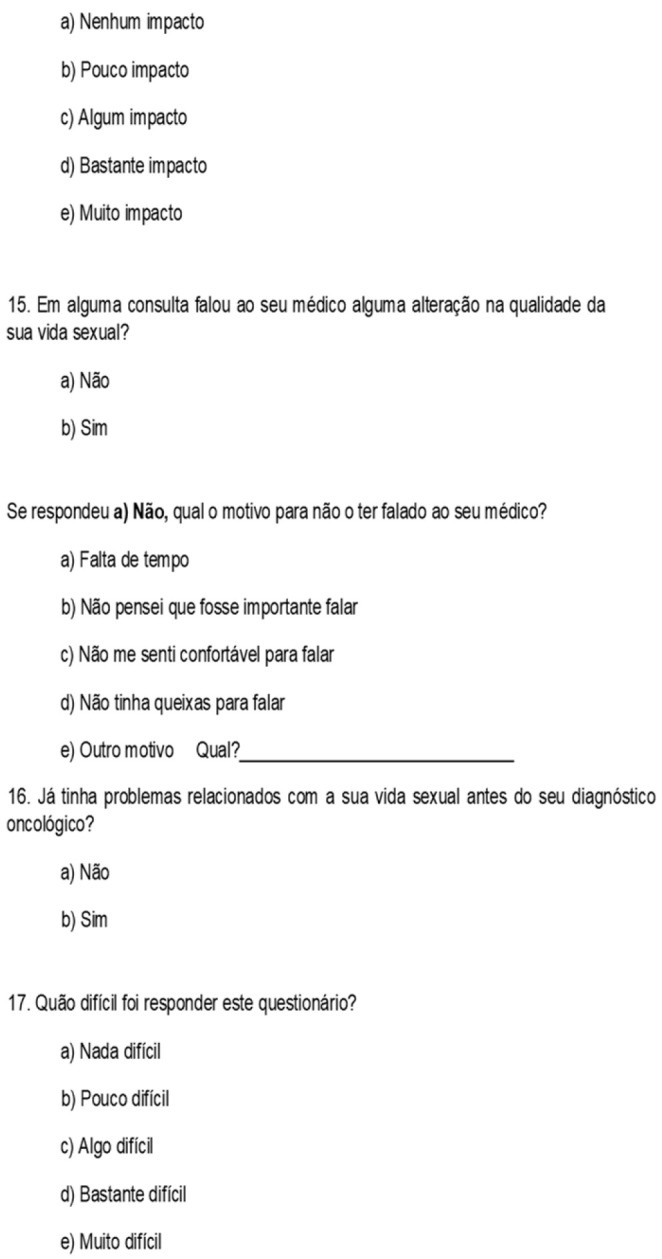
Page six.

**Figure A13:**
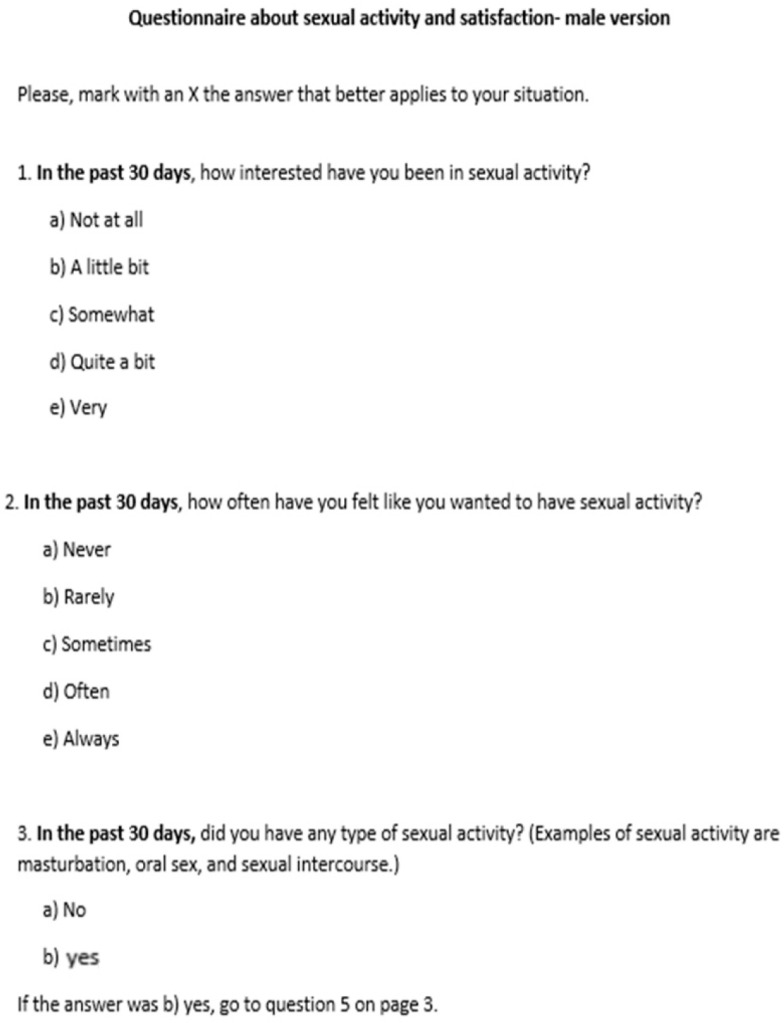
Page one.

**Figure A14:**
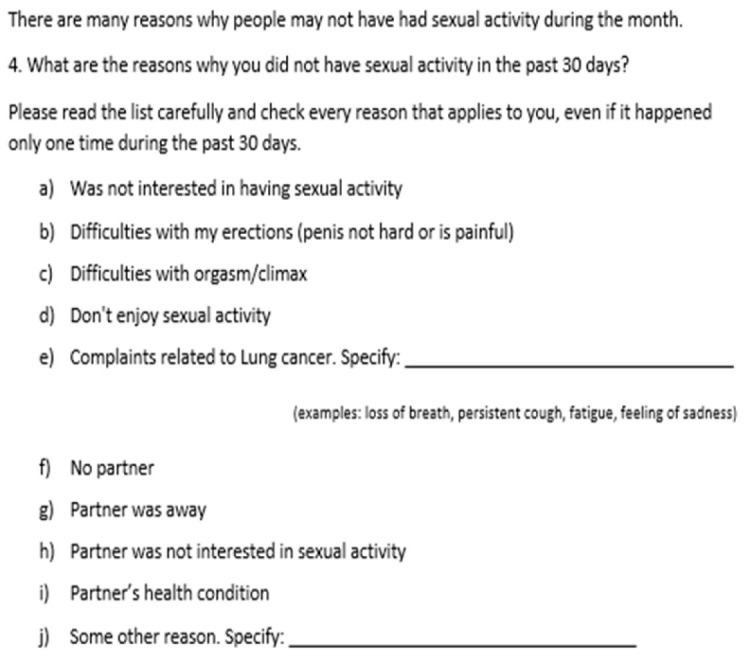
Page two.

**Figure A15:**
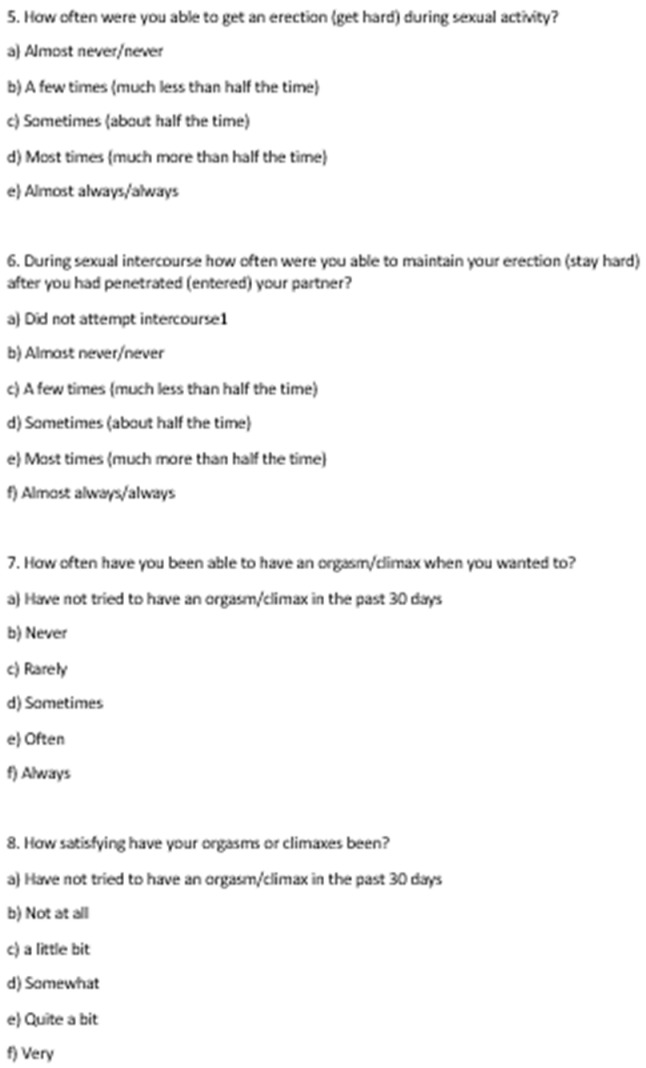
Page three.

**Figure A16:**
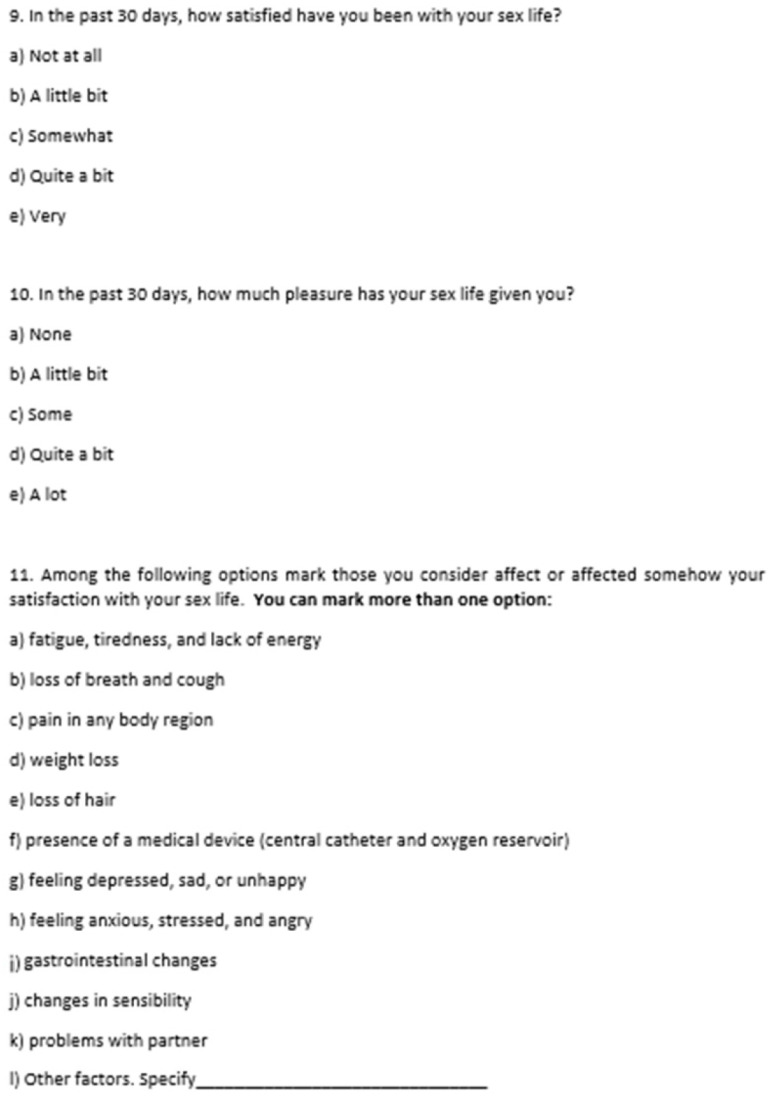
Page four.

**Figure A17:**
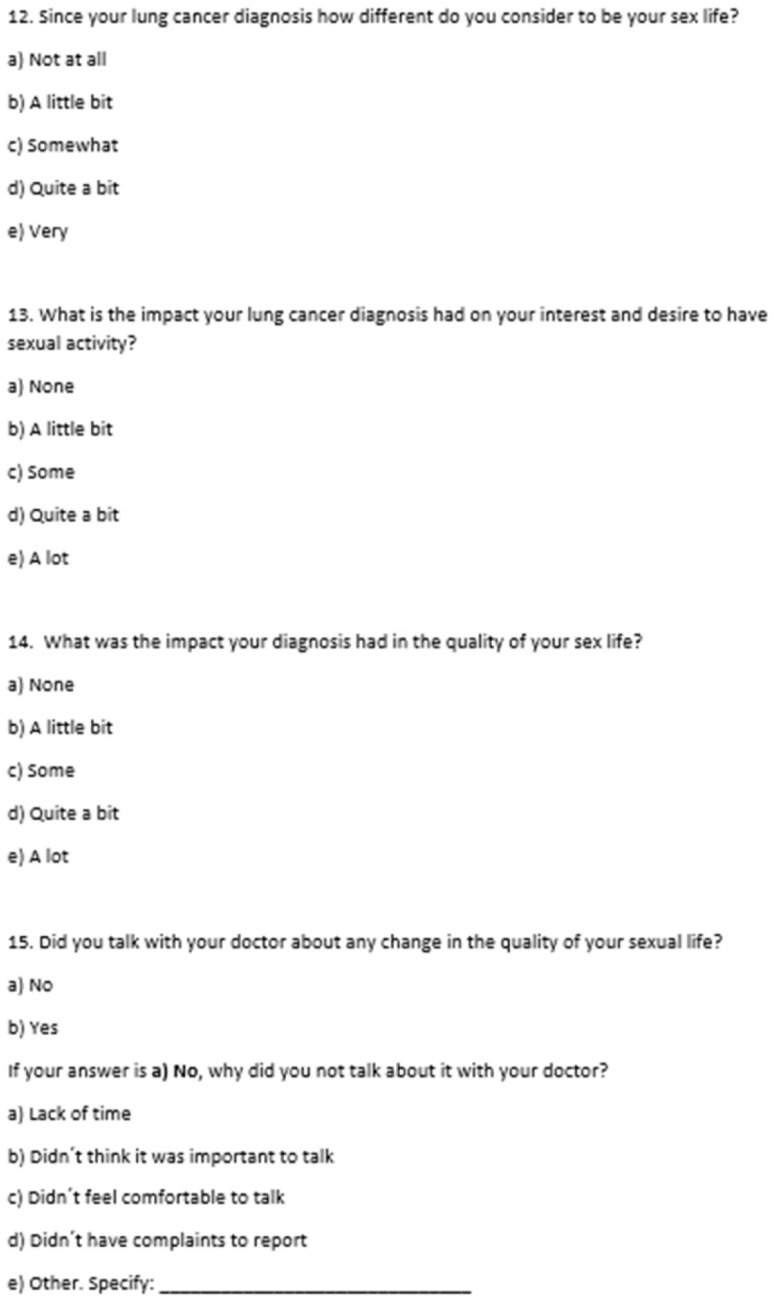
Page five.

**Figure A18:**
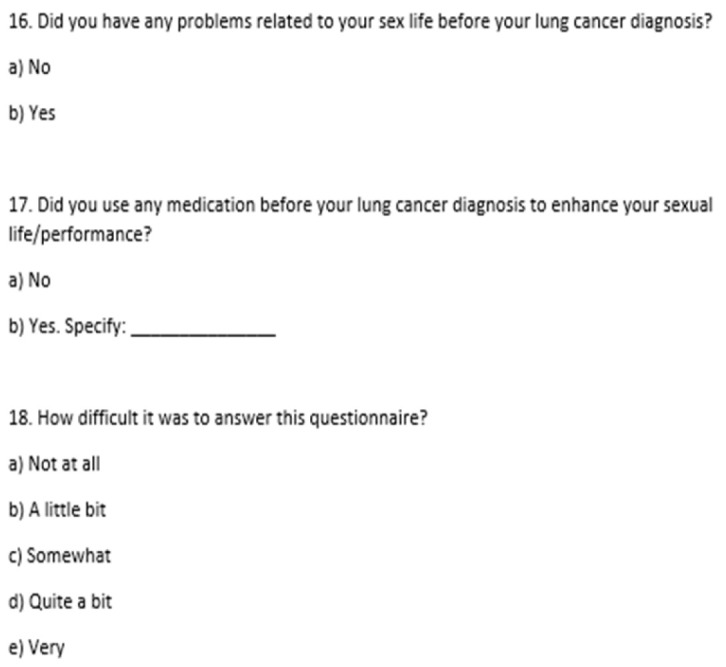
Page six.

**Figure A19:**
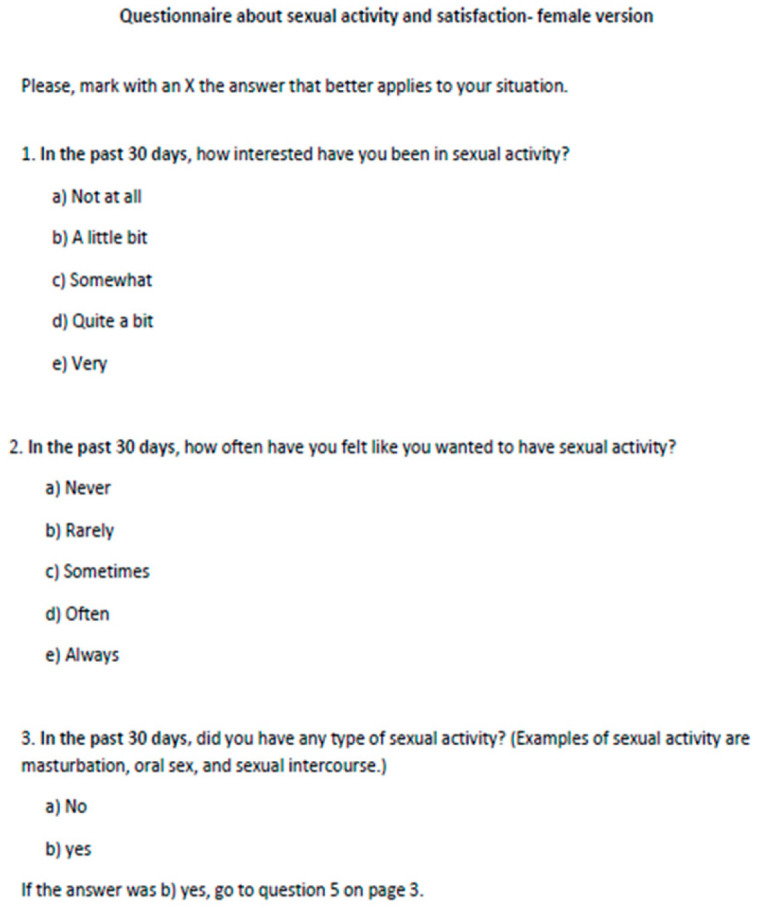
Page one.

**Figure A20:**
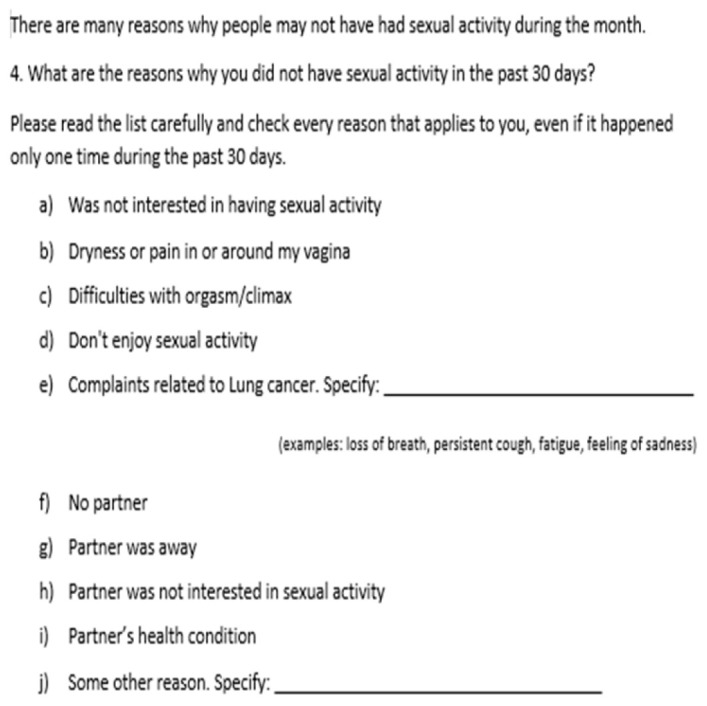
Page two.

**Figure A21:**
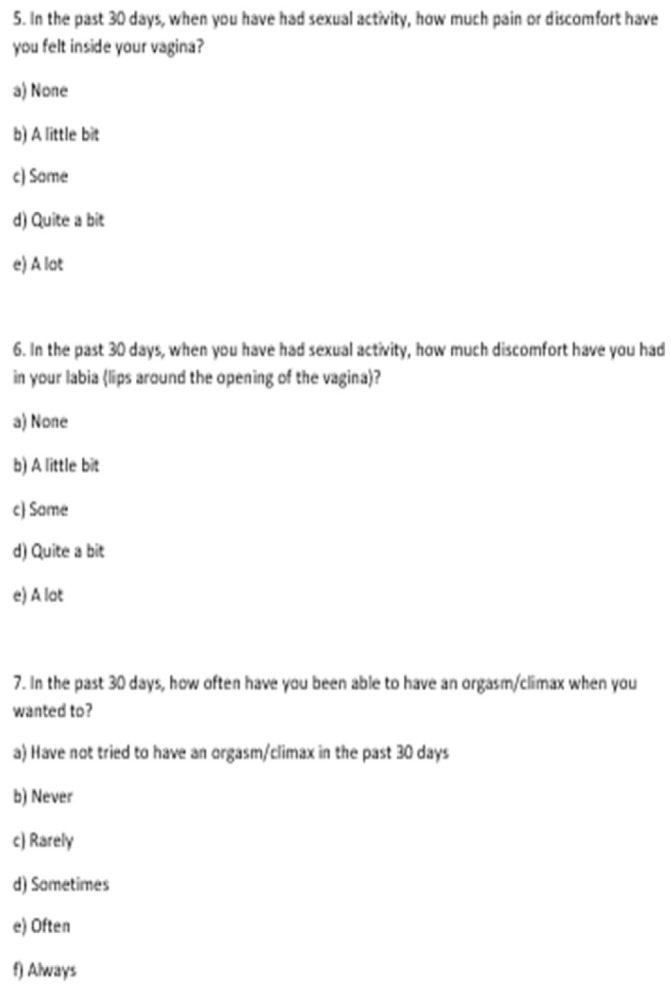
Page three.

**Figure A22:**
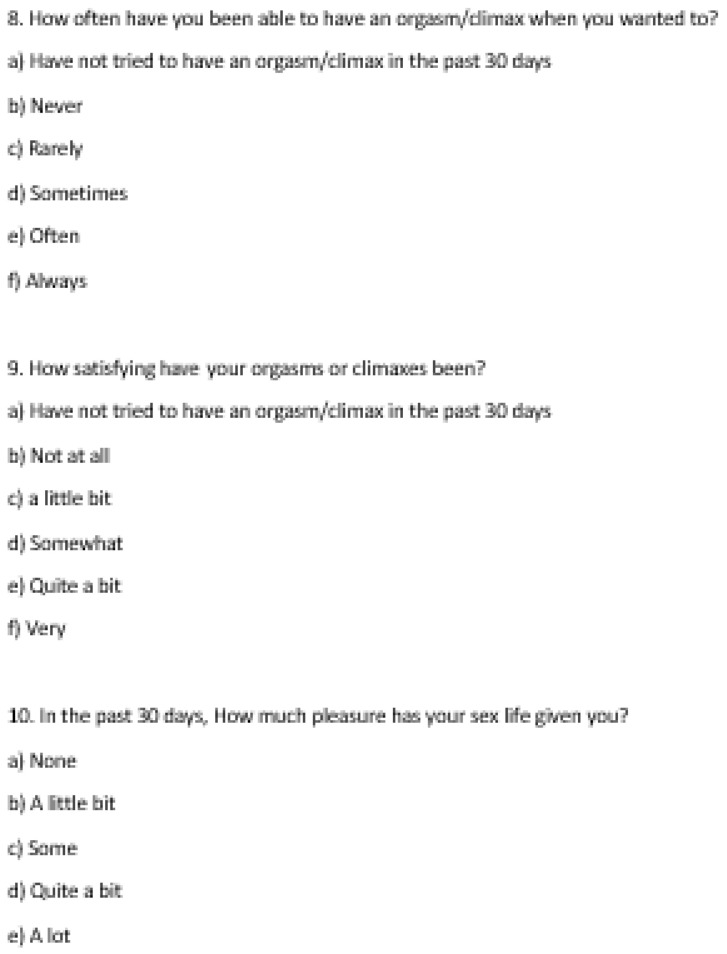
Page four.

**Figure A23:**
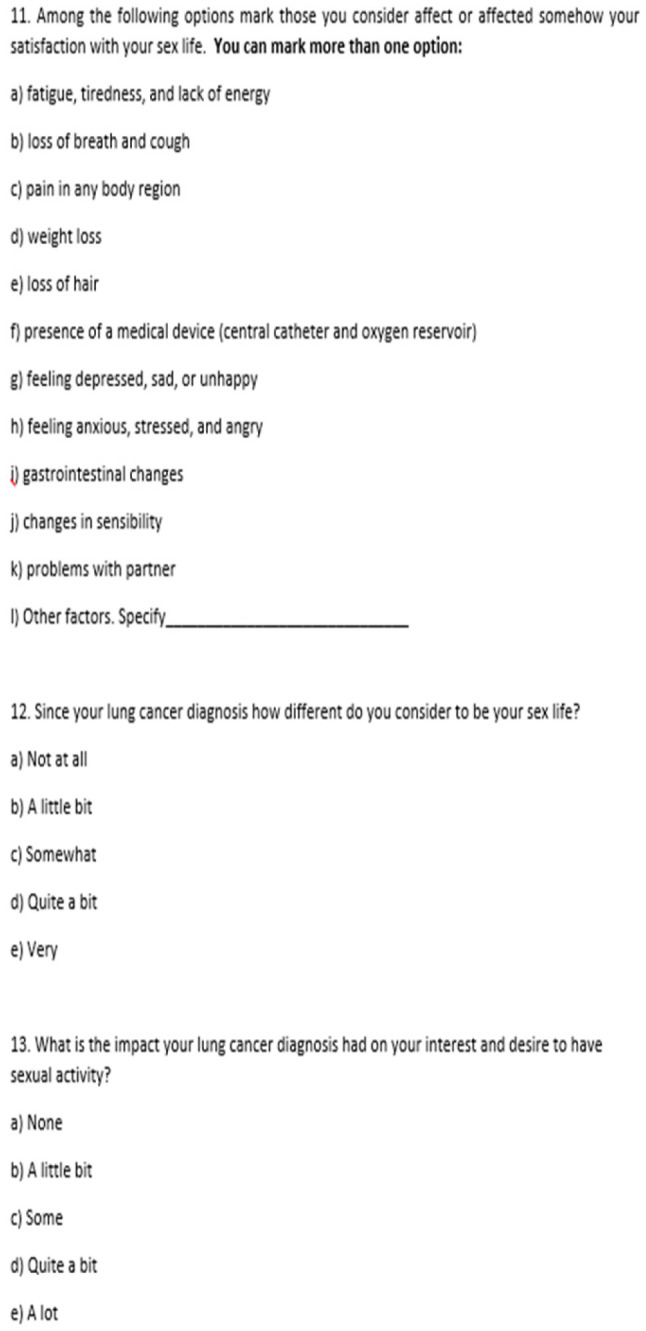
Page five.

**Figure A24:**
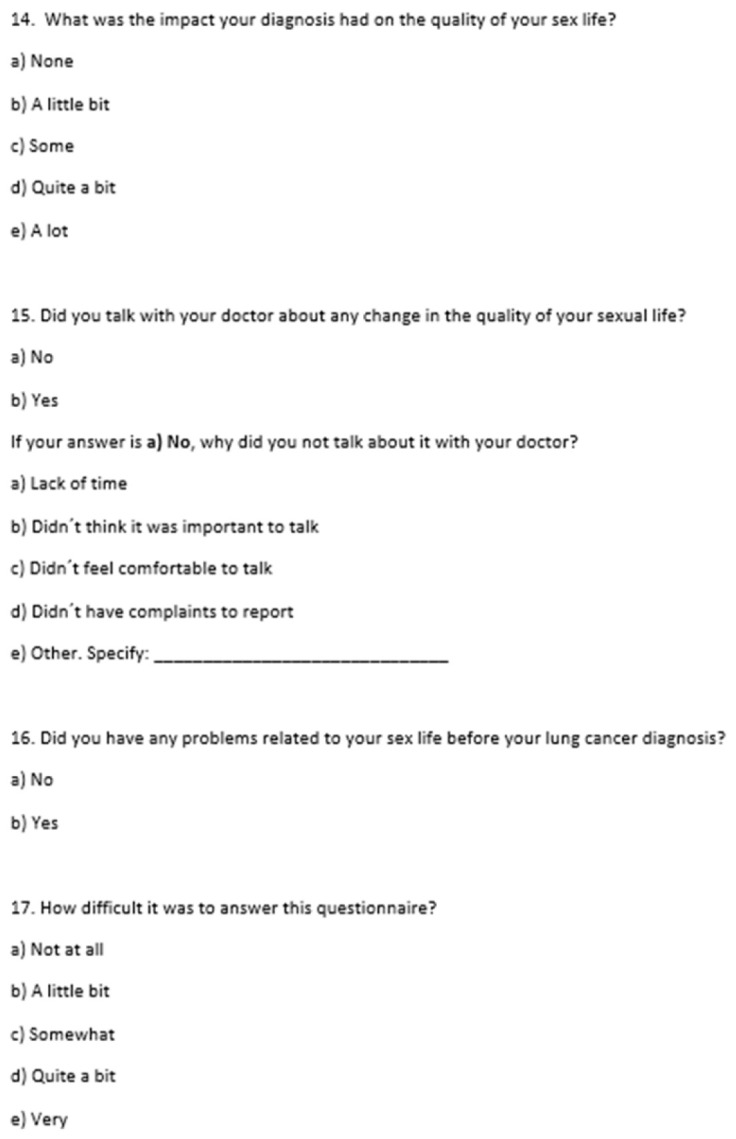
Page six.

**Figure A25:**
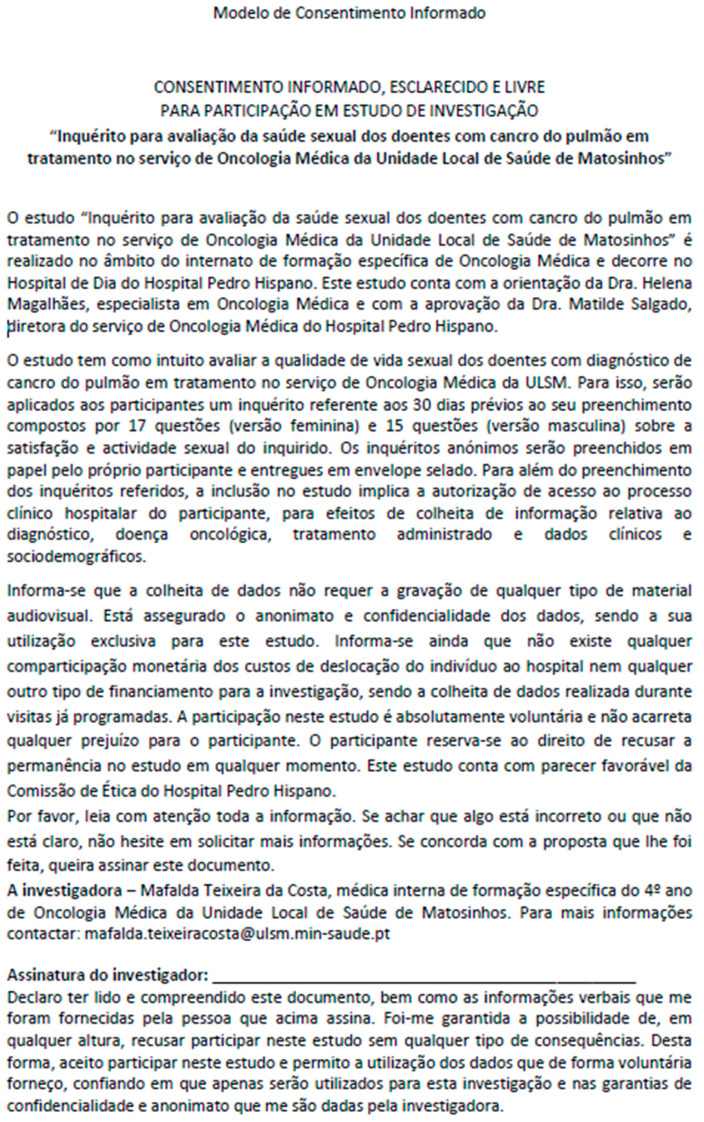
Page one.

**Figure A26:**
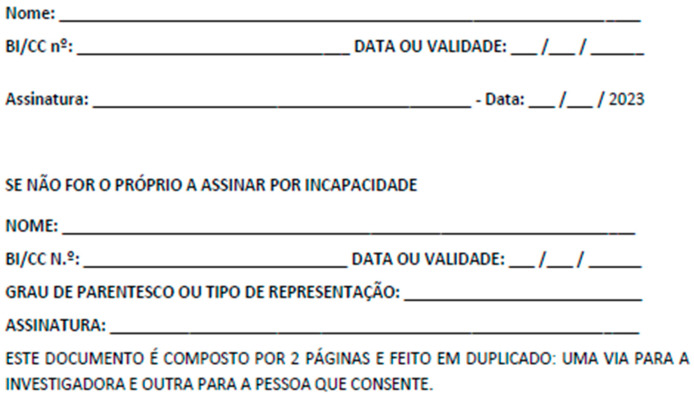
Page two.

## Data Availability

The data presented in this study are available upon request from the corresponding author due to privacy and ethical reasons.

## Abbreviations

The following abbreviations are used in this manuscript:

ECOG PS	Eastern Cooperative Oncology Group performance status
COPD	chronic obstructive pulmonary disease
NSCLC	Non-Small Cell Lung Cancer
NOS	not otherwise specified
SCLC	Small Cell Lung Cancer
nº	number
LC	lung cancer
TKIs	tyrosine kinase inhibitors
SRS	stereotactic radiosurgery
RT	radiotherapy
SBRT	stereotactic body radiation therapy
QRT	chemoradiotherapy
QT	chemotherapy
IT	immunotherapy
QTIT	chemoimmunotherapy
